# Fragile X and autism: Intertwined at the molecular level leading to targeted treatments

**DOI:** 10.1186/2040-2392-1-12

**Published:** 2010-09-21

**Authors:** Randi Hagerman, Gry Hoem, Paul Hagerman

**Affiliations:** 1 Department of Pediatrics, University of California, Davis, School of Medicine, Sacramento, California, USA; 2 MIND Institute, University of California, Davis, Health System, Sacramento, California, USA; 3 Molecular Cancer Research Group, Institute of Medical Biology, University of Tromso, Norway; 4 Department of Biochemistry and Molecular Medicine, University of California, Davis, School of Medicine, Davis, California, USA

## Abstract

Fragile X syndrome (FXS) is caused by an expanded CGG repeat (> 200 repeats) in the 5' untranslated portion of the fragile mental retardation 1 gene (*FMR1*), leading to deficiency or absence of the *FMR1 *protein (FMRP). FMRP is an RNA carrier protein that controls the translation of several other genes that regulate synaptic development and plasticity. Autism occurs in approximately 30% of FXS cases, and pervasive developmental disorder, not otherwise specified (PDD-NOS) occurs in an additional 30% of cases. Premutation repeat expansions (55 to 200 CGG repeats) may also give rise to autism spectrum disorders (ASD), including both autism and PDD-NOS, through a different molecular mechanism that involves a direct toxic effect of the expanded CGG repeat *FMR1 *mRNA. RNA toxicity can also lead to aging effects including tremor, ataxia and cognitive decline, termed fragile X-associated tremor ataxia syndrome (FXTAS), in premutation carriers in late life. In studies of mice bearing premutation expansions, there is evidence of early postnatal neuronal cell toxicity, presenting as reduced cell longevity, decreased dendritic arborization and altered synaptic morphology. There is also evidence of mitochondrial dysfunction in premutation carriers. Many of the problems with cellular dysregulation in both premutation and full mutation neurons also parallel the cellular abnormalities that have been documented in autism without fragile X mutations. Research regarding dysregulation of neurotransmitter systems in FXS, including the metabotropic glutamate receptor (mGluR)1/5 pathway and γ aminobutyric acid (GABA)_A _pathways, have led to new targeted treatments for FXS. Preliminary evidence suggests that these new targeted treatments will also be beneficial in non-fragile X forms of autism.

## Introduction

Fragile X syndrome (FXS) is an important subtype of autism, both because of its frequency and because knowledge of the molecular mechanisms involved in its pathogenesis has facilitated the development of targeted treatments with the potential to reverse or dramatically improve both behavioral and cognitive deficits. Because FXS is the most common single gene cause of autism, responsible for 2% to 6% of all cases of autism, it is clinically recommended that all individuals diagnosed with autism or ASD should have the FX DNA test (both PCR and Southern blot) when the etiology of their autism is not known [1-4]. FXS is nearly always caused by a trinucleotide (CGG) repeat expansion, located in the 5' untranslated region of the *FMR1 *gene, to a length of greater than 200 repeats (full mutation range). Full mutation expansions typically lead to methylation of the gene, reduced or absent transcription, and consequent decreased reduction in translation of the *FMR1 *protein (FMRP), the proximal basis of FXS. FMRP levels are correlated with the degree of clinical involvement including physical, cognitive and structural/functional brain involvement [5-10].

Approximately 30% of males with FXS have full autism, as determined by the standardized criteria of the Autism Diagnostic Observation Scale (ADOS) and the Autism Diagnostic Interview (ADI-R) [11-15]. An additional 30% of boys have pervasive developmental disorder, not otherwise specified (PDD-NOS) [11]. Among the remaining patients with FXS, of those who do not meet the criteria for an autism spectrum disorder (ASD) diagnosis, the majority have one or more autistic features, such as hand flapping, poor eye contact and tactile defensiveness [11].

A premutation CGG-repeat range (55 to 200 repeats) was initially defined in terms of an increased frequency of expansion of the CGG repeat to the full mutation range when transmitted by a premutation (carrier) woman. All children with the full mutation have a carrier mother, although a female patient with a premutation could have received this mutation from either her mother or her father. Moreover, the propensity for transmission of a full mutation allele increases with increasing CGG repeat number in the mother [16]. A father who is a carrier of either a premutation or full mutation allele will pass only a premutation to all of his daughters, presumably due to selective production of premutation allele-bearing sperm [17].

Carriers of premutation alleles were generally considered to be clinically uninvolved until premature ovarian failure, recently renamed FX-associated primary ovarian insufficiency (FXPOI), was reported [18]. Subsequently, the late onset neurodegenerative disorder, FX-associated tremor ataxia syndrome (FXTAS), was described [19,20], further establishing clinical involvement among premutation carriers. It is now evident that a spectrum of neurodevelopmental and aging/neurological problems are associated with premutations, including autism and ASD [21-26]. Most individuals with a premutation are neither developmentally disabled nor do they have autism; however, a subgroup does experience cognitive, emotional and/or behavioral involvement. There is a negative correlation between CGG repeat number and the level of FMRP in a premutation range [27], which predisposes individuals in the high end of a premutation range to cognitive and behavioral impairment. In addition, all individuals with a premutation have elevated *FMR1 *mRNA, whereas the opposite occurs in the full mutation [27]. Thus, the cognitive and behavioral impairments in a premutation and full mutation ranges are likely to have both distinct and overlapping mechanisms.

## Clinical and molecular involvement in FXS, and association with autism

**Figure 1:**
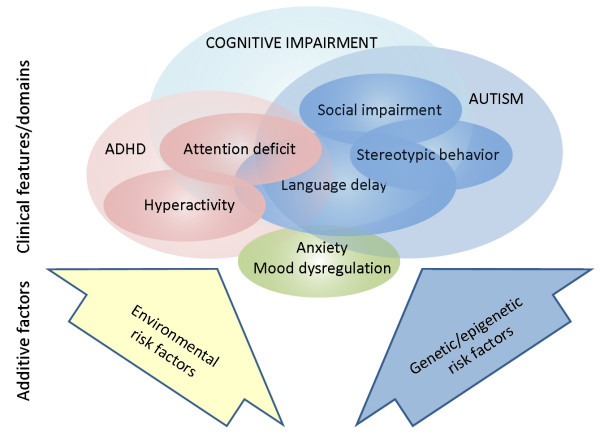
**Overview of the behavioral/cognitive phenotype of fragile X syndrome (FXS)**. The interrelationships among cognitive, behavioral and attentional deficits in FXS are modified by additional environmental influences and genetic background effects. Environmental influences include seizures, trauma, abuse and socioeconomic status. Genetic influences include allelic variations, additional genetic disorders and variation in the expression levels of genes important for the phenotype of FXS.

The basis for incomplete penetrance of autism (30%) or PDD-NOS (30%) among individuals with FXS is not known. However, there is evidence that patients with additional medical disorders that affect the CNS, such as seizures or additional genetic problems, have an increased risk for autism compared with patients with FXS alone [28-30]. For those with both FXS and autism, there is a spectrum of involvement with significant heterogeneity, both cognitively and behaviorally, with IQ values ranging from severely intellectually impaired to normal, particularly in females. However, there is a strong association between low IQ and the autism diagnosis in both males and females with FXS [11-14,31-35]. The cause of this heterogeneity is related to background genetic effects and environmental effects that influence IQ, social abilities, anxiety, attention deficit hyperactive disorder and additional features that are components of the FXS phenotype (Figure 1). Background genetic effects include additional pathological mutations (FXS has been reported with sex chromosome disorders, Down syndrome, Tourette syndrome and other conditions [28,29,36], allelic variants [37], and gene expression changes [38]). An example of the later condition is the Prader-Willi phenotype (PWP) of FXS, in which there is no structural or methylation change at 15q 11-13; rather, there is significant downregulation of expression of CYFIP 1, which is located at the 15q locus in Prader-Willi syndrome (PWS) [38]. Males with the PWP of FXS have severe obesity, hyperphagia and hypogenitalia, and a higher rate of ASD than those with FXS without the PWP [38].

Environmental influences on the phenotype of FXS include exposures to toxins (for example, alcohol, leading to fetal alcohol syndrome and FXS), abuse (physical or sexual), neglect, perinatal asphyxia, head trauma, seizures and socioeconomic status. Additional environmental exposures leading to further toxicity are just beginning to be explored in both premutation and full mutation involvement, as they are in idiopathic autism [39-42]. Such studies are occurring at a cellular level in premutation neurons; these neurons die earlier than do control neurons, with increased cell death documented by 21 days in culture [43]. In addition, mitochondrial dysfunction has been documented in fibroblasts and brain tissue in premutation carriers both with and without FXTAS [44]. We hypothesize that premutation neurons are more vulnerable to environmental toxins, and clinical case reports appear to support this notion [42,45].

**Figure 2:**
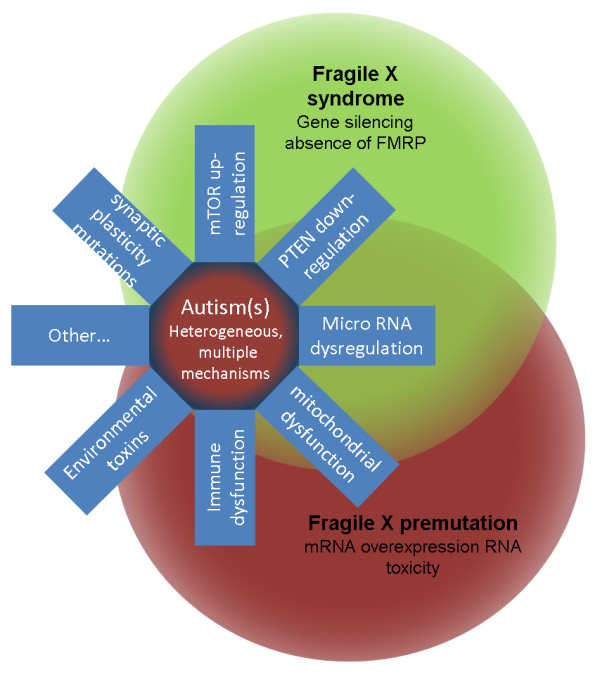
**Molecular overlap between autism, fragile x syndrome (FXS) and premutation disorders**. Absence of the FMR1 protein (FMRP) leads to the dysregulation of several proteins including those involved with synapse formation and plasticity, glutamate and γ aminobuyric acid (GABA) neurotransmission and mammalian target of rapamycin (mTOR) and phosphatase and tensin homolog (PTEN) pathways. A premutation is associated with elevation of *FMR1 *mRNA, leading to sequestration of proteins and mitochondrial dysfunction. Many of these same molecular changes can also occur in some types of autism. Some patients with FXS have mosaicism of premutation and full cells, so there is overlap of the molecular mechanisms among all three disorders.

The absence of FMRP in individuals with FXS has significant consequences in the translation of dozens and probably hundreds of proteins. Because FMRP usually suppresses translation, its absence leads to broad translational upregulation in the hippocampus [46]. Recent studies by Darnell *et al. *[47] and others have demonstrated linkage between FMRP and many proteins that are related to autism, including neuroligin 3 and 4, neurorexin, PDP (postsynaptic density protein) 95, CYFIP (cytoplasmic FMR1 interacting protein) 1 and 2, SHANK (Src homology 3 and multiple ankyrin repeat domains)3, Arc, PTEN (phosphatase and tensin homolog), MAPK (mitogen activated protein kinase), JAKMIP (janus kinase and microtubule interacting protein)1 and HERC (homologous to the E6-AP carboxyl terminus) and regulator of chromosome condensation (RCC)1-like domain-containing protein) 2, among others [2,4-50]. Most of these proteins are associated with synapse formation and plasticity; however, the *PTEN *gene encodes a dual specificity phosphatase effecting G1 cell cycle arrest and/or apoptosis, and 17% (3/18) of individuals with autism and macrocephaly were found to have a *PTEN *mutation [51]. Macrocephaly also occurs in FXS, often with a broad forehead remarkably similar to the broad foreheads described by Butler and colleagues; this characteristic is hypothesized to be related to the downregulation of PTEN that occurs in FXS [47,52]. Expression of JAKMIP1 and the G protein coupled receptor (GRP)155 were both altered by reduction of FMRP (seen in FXS) or induction of CYFIP1 (seen in the 15q duplication form of autism) *in vitro *[53]. These proteins were also dysregulated in boys with idiopathic ASD relative to their unaffected siblings [53]. Both CYFIP1 (a partner protein to FMRP and regulated by FMRP) and JAKMIP1 are involved with the RacGTPase system, which modulates the neurite development that is crucial for proper brain connectivity [54]. There is also evidence for upregulation of the mammalian target of rapamycin (mTOR) pathway in the hippocampus of the knockout (KO)mouse [55] and in studies of humans with FXS [56]. The mTOR pathway is dysregulated in several other genetic disorders that are associated with autism, such as tuberous sclerosis (TS) [57]. These findings have stimulated targeted treatments using rapamycin to downregulate the mTOR system in patients with TS, with initially positive results. The overlap of molecular mechanisms in those with a premutation or the full mutation and idiopathic autism is shown in Figure 2.

Recently, a number of studies have directly compared patients with FXS and those with autism without FXS. There are unique structural differences in the central nervous system (CNS) between the two disorders even when both disorders have comparable degrees of autism as assessed by standardized behavioral measures [58]. Those with FXS have an enlarged caudate compared with typically developing individuals and those with autism, whereas those with autism have a larger amygdala compared with FXS or controls [58]. These differences continue to evolve with age, as does the severity of the autistic features in FXS [59]. Therefore, from early in life, and probably *in utero*, there are structural CNS changes that are related to the lack of FMRP. The dysregulation of proteins that are important for synaptic plasticity and connectivity in the brain leads to the gradual deficits in socialization, behavior and cognition that characterize the FXS phenotype [60,61]. Although eye contact problems are usually not present during the first year of life, they evolve over time, as do the sensory hyperarousal, anxiety, motor and social deficits. Hoeft *et al. *[62] have reported that the early trajectory of brain growth abnormalities in FXS becomes more exaggerated over time and includes enhanced growth of the caudate, nucleus basalis and thalamus, compared with controls. Those authors also documented enhanced white matter volume, particularly of the striatal-frontal regions, becoming more dramatic in the early years (1 to 3 years of age), which suggests axonal pathology as opposed to secondary connectional dysregulation [62]. Their work further suggests that the earlier the intervention is begun, the better the outcome for an individual with FXS. These findings provide neurobiological support for initiating interventions as early in the lifespan as possible, although further clinical studies are needed. A summary of treatment for FXS was reviewed by Hagerman *et al. *[63].

## RNA toxicity and a premutation carrier

The discovery of the neurodegenerative disorder, FXTAS, in older adult carriers of premutation alleles, coupled with increased *FMR1 *transcriptional activity in the premutation range, led to the recognition of an entirely distinct pathogenic mechanism associated with the *FMR1 *gene: RNA toxicity [64-68]. A range of studies on the adverse consequences of expressing the expanded CGG repeat in human, animal and cell models has helped to establish an RNA toxicity model involving a toxic gain of function of premutation *FMR1 *RNA [69-79]. However, although carriers of premutation alleles have elevated *FMR1 *mRNA [27,80,81], the strongest argument for an RNA-based toxicity mechanism in both FXTAS and FXPOI [82-84], is that these clinical syndromes are limited to a premutation repeat range, where the gene is active; that is, low levels (or absence) of FMRP do not cause either FXTAS or FXPOI. However, moderately lowered FMRP levels in the upper premutation range may compound the effects of elevated RNA levels (a mechanistic issue that still needs to be resolved) but the primary effect appears to be expression of the expanded CGG-repeat RNA. A supporting argument for an RNA-based mechanism is that the *FMR1 *mRNA is found within the characteristic intranuclear neuronal and astrocytic inclusions of FXTAS [85,86].

FXTAS was originally described as a late adult-onset neurodegenerative disorder; however, there is an emerging view that FXTAS, and probably also FXPOI, is the end stage of a process that actually begins in early development, and that may be responsible for the emotional and behavioral problems, cognitive impairment, ASD and seizure activity experienced by children who are carriers of premutation alleles [21,25,87]. This view is based on a combination of animal and cell-based studies for early abnormalities resulting from expression of a premutation allele. In particular, Chen *et al. *[43] demonstrated that in cultured hippocampal neurons from day 1 postnatal premutation (knock-in; KI) mice, there were CGG repeat-dependent decreases in both the number of branches and the interbranch lengths, and decreased longevity in culture. Moreover, Garcia-Arocena *et al. *[88] observed abnormal lamin A/C architecture, with loss of ring-like nuclear staining, in embryonic fibroblasts from the KI mouse. In behavioral studies with the KI mice, there were progressive deficits in spatial processing (but no motor involvement) in mice as young as 12 weeks [70,89]. These observations, plus elevated levels of *FMR1 *mRNA in children with premutation alleles [90], support the presence of an early developmental component of *FMR1 *mRNA-associated toxicity.

Based on the toxic RNA gain of function model for myotonic dystrophy, in which disease pathogenesis involves the sequestration of one or more proteins by an expanded rCUG repeat in the 3' untranslated region of the myotonic dystrophy protein kinase (*DMPK*) gene [91,92], the first view of FXTAS envisioned a similar, direct-RNA mechanism in which proteins would be sequestered by the expanded CGG repeat [19,65,67,69]. A growing number of animal and cell- l-based studies support this 'direct RNA' model [71,72,93,94]. Recently, Sellier *et al. *[94]presented evidence for both sequestration of an RNA processing protein, Sam68 and the consequent altered splice-site regulation of several RNAs whose splicing is known to be regulated by Sam68. In addition to their demonstration of the functional consequences of Sam68 sequestration, Sellier *et al. *demonstrated that the incorporation of the protein into nuclear aggregates displayed a CGG-repeat cutoff that meant aggregation only occurred for expansions exceeding ~40 CGG repeats. More recently, Sellier *et al. *[95] also reported that a consequence of this sequestration is dysregulation of microRNAs, which may be related to the clinical problems of premutation carriers.

**Figure 3:**
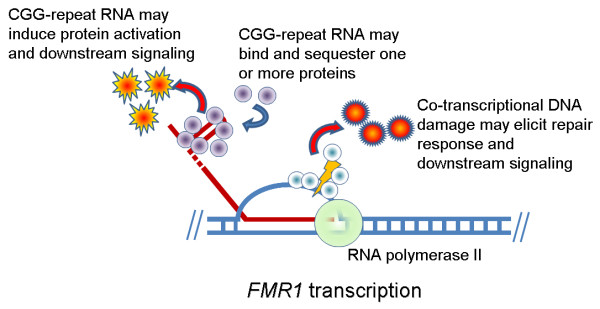
**Potential mechanisms of *FMR1 *mRNA toxicity**. Although numerous studies point to RNA toxicity as the underlying pathogenic trigger in fragile X-associated tremor ataxia syndrome (FXTAS), the specific mechanism for such toxicity is not known. Possibilities include (1) sequestration of one or more proteins that bind to the RNA, thus attenuating their other cell functions; (2) protein activation upon binding to the CGG-repeat RNA, leading to dysregulation of one or more signaling cascades; and (3) various co-transcriptional process, such as R-loop formation, that lead to DNA damage/repair signaling and consequent cellular dysregulation.

It should be noted that although the sequestration model remains the most viable mechanism for RNA toxicity, the clinical data only support the requirement for transcription. Thus, a role for other mechanisms such as RNA-triggered signaling or co-transcriptional mechanisms cannot be discounted [68] (Figure 3). Evidence for a direct RNA-based (for example, sequestration) model cannot exclude the possibility that co-transcriptional RNA, or even DNA, has a role in the pathogenesis. Entezam and Usdin [74] observed that the DNA-repair protein ATR is recruited to CGG expansions, and the fact that another DNA-repair protein, γ-H2AX[96], is found in the intranuclear FXTAS inclusions [97], suggests that transcription-induced DNA damage could also trigger the pathogenesis of premutation-associated disorders.

Recent work from the laboratory of Guilivi has demonstrated mitochondrial dysfunction in fibroblasts and brain samples in premutation carriers both with and without FXTAS [44]. Mitochondrial dysfunction in carriers included uncoupling between electron transport and synthesis of ATP in addition to decreased levels of mitochondrial proteins including the ATPase β-subunit (ATPB) from complex V, the cytochrome c oxidase subunit from complex IV (CCOIV) and manganese superoxide dismutase as part of the mitochondrial antioxidant defense. The levels of the mitochondrial proteins correlated inversely with the CGG repeat numbers in the premutation range. These protein changes increased oxidative stress and oxidatively modified mitochondrial proteins, and activated the unfolded protein response and phosphorylation of the alpha subunit of the heterotrimeric eukaryotic translational initiation factor 2 (eIF2α), resulting in a decrease in protein translation. Similar types of mitochondrial abnormalities have been seen in those with autism without a FX mutation (Giulivi *et al. *unpublished data) [98,99]. Specifically, Olivera *et al. *[98] reported that 14 of 69 patients with autism had hyperlactacidemia, and in 5 of 11 of these patients who underwent a deltoid muscle biopsy, there was a mitochondrial respiratory chain disorder with enzyme function that was <20% of normal mean activity, including complex I, complex IV and complex V abnormalities. Weissman *et al. *studied 25 patients with autism and evidence of oxidative phosphorylation abnormalities, and found 19 with elevated lactate levels, 64% with complex I deficiency and 20% with complex III deficiency. Two of the patients had pathological mitochondrial DNA mutations [99]. Other reports of mitochondrial gene mutations in children with autism have also been reported [100-102].

## Clinical involvement of some premutation carriers

Although autism and other clinical involvement in a subgroup of young premutation carriers was initially thought to be only an occasional occurrence [26,103-106], research cohorts demonstrated that approximately 14% of boys and 5% of girls with a premutation had ASD [107]. More recent studies demonstrated a high rate of ASD (73%) in boys with a premutation who were referred clinically to the UC Davis MIND Institute, although this was much lower in premutation males who were identified by cascade testing (7%) compared with their brothers who did not have a premutation (0%) [108]. Although there is clearly a bias towards an ASD phenotype in those who present clinically, a recent online family questionnaire completed by over 1200 families affected by FXS found that 19% of 57 males with a premutation had a diagnosis of autism, which was significantly higher than control boys (5%). In this survey 1% of 199 females with a premutation also had a diagnosis of autism [87]. This same survey found that 33% of boys with a premutation had developmental delays, which was significantly higher than in a group of age-matched boys without a premutation (1.8%). A completely unbiased population of premutation carriers that should be followed carefully are those diagnosed by screening as newborns; three studies are currently in progress in the USA.

Studies of neuropsychological deficits in premutation carriers during adulthood have been complicated because of the subclinical CNS changes that can occur related to the development of FXTAS [109-111]. Studies have detected deficits in executive function in a subgroup of males with a premutation, but not in the corresponding group of females [112-117]. In contrast to these four reports, Hunter *et al. *[118] found no neuropsychological deficits in 54 men with a premutation who were aged under 50 years, although the Behavioral Dyscontrol Scale (BDS) [113,119], which was found to be most sensitive to executive dysfunction in older male carriers [112], was not used. Clearly, recruitment bias is likely to affect adult premutation studies in neuropsychological testing and in emotional assessments. In contrast to the neuropsychological testing, standardized emotional assessments have demonstrated problems with anxiety and/or depression in both males and females with a premutation, both with and without FXTAS, compared with controls at multiple centers [22,120-124]

An emerging phenotype includes the finding of autoimmune problems in a subgroup of women with a premutation. These problems include fibromyalgia, hypothyroidism and multiple sclerosis, and they can occur in women with a premutation both with and without FXTAS [24,125-127]. Hunter *et al. *[82] found that women with irregular cycles reported higher rates of thyroid disease in addition to depression/anxiety. The molecular process leading to the autoimmune problems are unknown, although they are most likely related to the RNA toxicity. Predisposing factors leading to autoimmune disease in some females are likely to be genetic, because in our clinical experience, they cluster in families. Because of concern about the genetic factors that underlie both autoimmune disease and autism, we studied whether there is an increase in ASD with FXS in the children of female carriers who have autoimmune disease, compared with carriers who do not have autoimmune disease [128]. The odds ratio (OR) for ASD was 1.27 (*P *= 0.51) which was not significant; however, the ORs for seizures and tics in the offspring were 3.81 (*P *= 0.031) and 2.94 (*P *= 0.019) respectively. These results raise the possibility that there are intergenerational autoimmune factors or perhaps auto-antibodies that affect the prevalence of seizures and tics in the offspring of mothers with a premutation and autoimmune disease [128].

## FMRP function throughout life leading to targeted treatments for FXS

FMRP is an mRNA-binding protein that is important for mRNA transport, mRNA stabilization and translation of mRNA into protein at the synapse [129-131]. FMRP is also a factor in the regulation of adult neurogenesis, so in the absence of FMRP there is dysregulation of glycogen synthase kinase (GSK)3β, reduced β-catenin and defective Wnt signaling. These alterations lead to downregulation of neurogenin 1, which is an early initiator of neuronal differentiation and an inhibitor of astrocyte differentiation [132]. Therefore, FMRP is important throughout life and there is a high incidence of motor problems, including Parkinson disease (PD), with aging in those with FXS [133]. In addition, in neuropathologic studies, there is evidence of migration problems in the hippocampus and in the cerebellum in those with FXS (Greco *et al*,. unpublished data), which are similar to those reported in individuals with autism [134]. These problems may be related to dysregulation of Wnt signaling in both FXS and autism.

Perhaps the most important change in protein expression in the absence of FMRP is the excess basal translation of proteins involved in the metabotropic glutamate receptor (mGluR) 5 receptor pathway [135]. Bear and colleagues have proposed the mGluR theory of FX, suggesting that the deficits associated with FXS are related to upregulation of the downstream effectors of the mGluR5 pathway, leading to enhanced long-term depression (LTD), and that treatment with an mGluR5 antagonist could be a targeted treatment for FXS [135,136]. Both FMRP and mGluRs play important roles in synaptogenesis and synaptic plasticity, and in the absence of FMRP there are long, thin and immature dendritic spines in both human and animal models of FXS [137-142]. There are also enhanced, abnormal epileptiform discharges consistent with an enhanced rate of clinical seizures in FXS [143,144].

Support for the 'mGluR theory' has been shown by generating *FMR1 *mutant mice with a 50% reduction in mGluR5 expression [145]. The mGluR5 deficiency rescued most of the KO mouse abnormalities including altered ocular dominance plasticity, increased density of dendritic spines on cortical pyramidal neurons, increased basal protein synthesis in the hippocampus, exaggerated inhibitory avoidance extinction, audiogenic seizures and accelerated body growth. However, macroorchidism was not rescued. This work is supportive of the proposal by Bear *et al. *[146]that excessive mGluR5 signaling is responsible for the psychiatric and neurological symptoms of FXS, including cognitive deficits, seizures, anxiety, perseverative movements and social deficits.

Use of mGluR5 antagonists in animal models of FXS further supports the mGluR theory. MPEP (2-methyl-6-phenylethynyl pyridine hydrochloride) is a potent, highly selective antagonist of mGluR5 receptors [147]. *In vitro*, both MPEP and fenobam, another mGluR5 antagonist, were able to rescue hippocampal dendritic abnormalities in the KO mice [148,149]. MPEP has reversed audiogenic seizures, epileptiform discharges, open field hyperactivity and the defect in prepulse inhibition (PPI) of the startle response in KO mice [148-150]. When MPEP and lithium, a partial mGluR5 antagonist that also blocks GSK3β, were given to *dfmr1 *loss of function *Drosophila *mutants, the flies had restored normal courtship behavior, memory and brain structural abnormalities through the reduction of mGluR activity [151]. MPEP is toxic to humans, so other mGluR5 antagonists including fenobam have been studied in FXS [152,153]. Fenobam was found to be safe in a single dose trial in 12 adults with FXS. There were improvements in hyperactivity and anxiety, and 50% showed at least a 20% improvement in PPI [152]. Currently there are two additional mGluR5 antagonists undergoing trials in adults with FXS at multiple centers [153].

Other mechanisms to downregulate glutamate release and modulate mGluR overactivity have been investigated. γ Aminobutyric acid (GABA)_B _receptor agonists, such as baclofen, inhibit both presynaptic release of glutamate and postsynaptic transmission and/or intracellular signaling downstream from mGluR5 [154-156]. Baclofen has been shown to be efficacious in treating hyperactivity [157], marble burying (Seaside Therapeutics, unpublished data) and audiogenic seizure phenotypes in FX KO mice [158]. A double-blind, placebo-controlled, crossover trial of arbaclofen, the right sided isomer of baclofen that is significantly more potent than regular baclofen as a GABA agent, has just been completed at multiple centers,and involved over 60 individuals with FXS (aged 6 years and older). The preliminary safety and efficacy results are positive, with improvement in the Clinical Global Impression Improvement scale in those with the most severe baseline ratings [159]. There are also preliminary studies that are taking place involving individuals with autism without FXS, and these studies have also produced preliminary positive results. Therefore, further studies on both FXS and autism are set to take place.

The GABAergic system is also dysregulated in FXS, and GABA agents are important to consider for targeted treatment studies in FXS. GABA is a major inhibitory neurotransmitter receptor in the brain, which is important in anxiety, depression, epilepsy, insomnia, and learning and memory [160]. GABA-mediated inhibition is important for terminating ictal discharges and the spread of hyperexcitability, which can lead to seizures [161].

There are two main subtypes of GABA receptors: GABA_A _and GABA_B_. The main difference between them is that the first is a ligand gated Cl^- ^channel that gives fast inhibition, whereas the latter is a G-protein coupled receptor which gives slower and more prolonged inhibitory signals [162,163]. The metabotropic GABA_B _receptor can either be presynaptic and inhibit the release of neurotransmitters through downregulation of high-voltage activated Ca^2+^-channels; or, when postsynaptic, decrease neuronal excitability through its influence on K^+ ^channels. Thus, GABA_B _agonists such as arbaclofen mediate their downregulating effects on either side of the synapse. The ionotropic GABA_A _receptor is usually localized postsynaptically, and their activation leads to opening of Cl^- ^channels and hyperpolarization of the membrane potential, thus making it difficult for excitatory neurotransmitters such as glutamate to generate an action potential. GABA_A _receptors are more abundant than GABA_B _receptors in mammalian brain, and disorders such as epilepsy, sleep disorders and anxiety are now being treated using drugs that act on the GABA_A _receptor[164].

Direct binding between FMRP and the mRNA of the delta subunit of the GABA_A _receptor has been shown [165]. Reduced expression and dysfunction of several subunits of the GABA_A _receptor (α1, α3, α4; β1, β2; γ1, γ2 and δ) have been shown in FX animal models [166-168]. *FMR1 Drosophila *mutants destined to die from glutamate toxicity were rescued after administering molecules involved in the GABAergic pathway [166]. In addition, abnormal male courtship behavior and mushroom body abnormalities were rescued by GABA agents [166].

There is a profound reorganization of neocortical inhibitory circuits of GABAergic intraneurons in the KO mouse [164,167-173]. Recent evidence indicates that deficits in GABA-mediated inhibition may underlie many of the key symptoms in FXS, including the seizures, anxiety and autistic-like behaviors [167,169,173]. The neocortex in KO mice exhibits a marked reduction in the density of GABAergic interneurons that stain with parvalbumin. Moreover, electrophysiological studies in brain slices from these animals exhibit impaired GABA_A _receptor-mediated inhibitory function [174]. In addition to a gross reduction in GABA-mediated inhibition caused by the maldevelopment of inhibitory circuits and the loss of GABAergic interneurons, there is also evidence of altered GABA_A _receptor subunit expression in the FX KO mouse [167]. In particular, there appears to be a selective reduction in the expression of δ subunits [167,168]. Global expression analysis by means of the differential display in the FX mouse model revealed consistent underexpression of only three genes, one of which was the GABA_A _receptor subunit δ. As GABA_A _receptors are the major inhibitory receptors in the brain, and are specifically involved in processes that are disturbed in FX, including neuronal excitability (leading to enhanced seizure susceptibility), anxiety, sleep and learning, enhancement of the function of GABA_A _receptors may have major therapeutic benefits for FXS. Kooy and colleagues [175] have demonstrated that use of the GABA_A _agonist ganaxolone (3α-hydroxy-3β-methyl-5α-pregnan-20-one) improved seizures in the KO mouse model of FXS. Ganaxolone is a 3β-methylated synthetic analog of the progesterone metabolite allopregnanolone, which is itself a neuroactive steroid. Unlike progesterone, neither allopregnanolone nor ganaxolone have direct hormonal activity via progesterone receptor activation, and cannot cause hormonal side-effects. However, allopregnanolone and ganaxolone are powerful positive allosteric modulators of GABA_A _receptors [161]. Human trials indicate that ganaxolone is well tolerated and that it may be efficacious in the treatment of diverse forms of epilepsy in children and adults [176-180]. Plans for studies on ganaxolone are currently underway in children and adults with FXS.

Minocycline, a widely used antibiotic used to treat acne and skin infections, is another promising drug that may target core symptoms of FXS and autism. Minocycline inhibits matrix metalloproteinase (MMP)-9 and reduces inflammation in the central nervous system. MMPs are enzymes involved in synaptic plasticity, and are associated with immature dendritic spine morphology [140,181]; MMP-9 is elevated in FXS. When minocycline was administered to *FMR1 *KO mice, their hippocampal neurons exhibited mature dendritic spines, and behaviorally, they showed decreased anxiety and improved exploration skills [140]. Off-label use of minocycline to treat 50 individuals with FXS resulted in two-thirds of families noticing positive improvements in their child's language, attention and/or behavioral improvements while on the medication [182]. An open-label trial is ongoing to investigate the effects of minocycline on children with regressive autism at the National Institute of Mental Health (NIMH). Paribello reported beneficial effects on the CGI and the Aberrant Behavior checklist in an open trial of minocycline involving patients with FXS who were aged 13 and older [183]. Currently, a double-blind, placebo-controlled clinical trial is in progress at the Medical Investigation of Neurodevelopmental Disorders (MIND) Institute for individuals with FXS who are aged 3.5 to 16 years

FXS has led the way for targeted treatments in neurodevelopmental disorders, and many of the treatments guided by molecular abnormalities in FXS may also be helpful for non-FX autism. The treatment trials will now combine targeted treatments, which strengthen synaptic connections, with enhanced educational and behavioral interventions to further develop appropriate synaptic connections in FXS. These targeted treatments combined with educational interventions look promising for reversing the intellectual and behavioral problems of FXS. Because of the shared neurobiological and molecular pathways, these interventions will hopefully also prove helpful in a subset of patients with idiopathic autism

## Conclusions

FX syndrome and autism are intertwined, because FMRP regulates the translation of many messages that affect synaptic plasticity and connectivity in the central nervous system. The absence of FMRP also leads to upregulation of mGluR5 pathways and downregulation of GABA_A _pathways. Targeted treatments to reverse these problems are currently being studied in patients with FXS. Many of these targeted treatments may also be helpful for ASD without FXS.

A premutation can also cause ASD, particularly in a subset of young males, and the mechanism of involvement relates to elevated mRNA levels causing dysregulation of numerous proteins, early neuronal cell death in culture, mitochondrial dysfunction and vulnerability to environmental toxicity. Targeted treatments are currently being developed for premutation involvement in early childhood, and also for neurodegenerative problems including FXTAS in aging individuals.

## Competing interests

RH has received funding from Seaside Therapeutics, Novartis, Roche, Forest, Johnson & Johnson and Curemark for clinical trials, and also consults with Novartis and Roche regarding clinical trials in fragile X syndrome. PH is an unpaid consultant with Asuragen, and has a filed patent application for an *FMR1 *genotyping method. GH has no conflicts of interest.

## Authors' contributions

All authors helped draft the manuscript, and all authors read and approved the final manuscript.

## References

[B1] Miller DT Adam MP Aradhya S Biesecker LG Brothman AR Carter NP Church DM Crolla JA Eichler EE Epstein CJ Faucett WA Feuk L Friedman JM Hamosh A Jackson L Kaminsky EB Kok K Krantz ID Kuhn RM Lee C Ostell JM Rosenberg C Scherer SW Spinner NB Stavropoulos DJ Tepperberg JH Thorland EC Vermeesch JR Waggoner DJ Watson MS Martin CL Ledbetter DH Consensus statement: chromosomal microarray is a first-tier clinical diagnostic test for individuals with developmental disabilities or congenital anomalies Am J Hum Genet 2010 86 749 764 10.1016/j.ajhg.2010.04.006 20466091 PMC2869000

[B2] Nishimura Y Martin CL Vazquez-Lopez A Spence SJ Alvarez-Retuerto AI Sigman M Steindler C Pellegrini S Schanen NC Warren ST Geschwind DH Genome-wide expression profiling of lymphoblastoid cell lines distinguishes different forms of autism and reveals shared pathways Hum Mol Genet 2007 16 1682 1698 10.1093/hmg/ddm116 17519220

[B3] Reddy KS Cytogenetic abnormalities and fragile-X syndrome in Autism Spectrum Disorder BMC Med Genet 2005 6 3 10.1186/1471-2350-6-3 15655077 PMC548305

[B4] van Karnebeek CD Jansweijer MC Leenders AG Offringa M Hennekam RC Diagnostic investigations in individuals with mental retardation: a systematic literature review of their usefulness Eur J Hum Genet 2005 13 6 25 10.1038/sj.ejhg.5201279 15523501

[B5] Lightbody AA Reiss AL Gene, brain, and behavior relationships in fragile X syndrome: evidence from neuroimaging studies Dev Disabil Res Rev 2009 15 343 352 10.1002/ddrr.77 20014368 PMC4354896

[B6] Tassone F Hagerman RJ Ikle DN Dyer PN Lampe M Willemsen R Oostra BA Taylor AK FMRP expression as a potential prognostic indicator in fragile X syndrome Am J Med Genet 1999 84 250 261 10.1002/(SICI)1096-8628(19990528)84:3<250::AID-AJMG17>3.0.CO;2-4 10331602

[B7] Miller LJ McIntosh DN McGrath J Shyu V Lampe M Taylor AK Tassone F Neitzel K Stackhouse T Hagerman RJ Electrodermal responses to sensory stimuli in individuals with fragile X syndrome: a preliminary report Am J Med Genet 1999 83 268 279 10.1002/(SICI)1096-8628(19990402)83:4<268::AID-AJMG7>3.0.CO;2-K 10208160

[B8] Loesch DZ Huggins RM Hagerman RJ Phenotypic variation and FMRP levels in fragile X Ment Retard Dev Disabil Res Rev 2004 10 31 41 10.1002/mrdd.20006 14994286

[B9] Gothelf D Furfaro JA Hoeft F Eckert MA Hall SS O'Hara R Erba HW Ringel J Hayashi KM Patnaik S Golianu B Kraemer HC Thompson PM Piven J Reiss AL Neuroanatomy of fragile X syndrome is associated with aberrant behavior and the fragile X mental retardation protein (FMRP) Ann Neurol 2008 63 40 51 10.1002/ana.21243 17932962 PMC2773141

[B10] Hoeft F Hernandez A Parthasarathy S Watson CL Hall SS Reiss AL Fronto-striatal dysfunction and potential compensatory mechanisms in male adolescents with fragile X syndrome Hum Brain Mapp 2007 28 543 554 10.1002/hbm.20406 17437282 PMC6871315

[B11] Harris SW Hessl D Goodlin-Jones B Ferranti J Bacalman S Barbato I Tassone F Hagerman PJ Herman H Hagerman RJ Autism profiles of males with fragile X syndrome Am J Ment Retard 2008 113 427 438 10.1352/2008.113:427-438 19127654 PMC2629645

[B12] Rogers SJ Wehner EA Hagerman RJ The behavioral phenotype in fragile X: Symptoms of autism in very young children with fragile X syndrome, idiopathic autism, and other developmental disorders J Dev Behav Pediatr 2001 22 409 417 11773805 10.1097/00004703-200112000-00008

[B13] Kaufmann WE Cortell R Kau AS Bukelis I Tierney E Gray RM Cox C Capone GT Stanard P Autism spectrum disorder in fragile X syndrome: communication, social interaction, and specific behaviors Am J Med Genet 2004 129A 225 234 10.1002/ajmg.a.30229 15326621

[B14] Hatton DD Sideris J Skinner M Mankowski J Bailey DB Jr Roberts JE Mirrett P Autistic behavior in children with fragile X syndrome: prevalence, stability, and the impact of FMRP Am J Med Genet A 2006 140 1804 1813 10.1002/ajmg.a.31286 16700053

[B15] Hall SS Lightbody AA Reiss AL Compulsive, self-injurious, and autistic behavior in children and adolescents with fragile X syndrome Am J Ment Retard 2008 113 44 53 10.1352/0895-8017(2008)113[44:CSAABI]2.0.CO;2 18173299

[B16] McConkie-Rosell A Abrams L Finucane B Cronister A Gane LW Coffey SM Sherman S Nelson LM Berry-Kravis E Hessl D Chiu S Street N Vatave A Hagerman RJ Recommendations from multi-disciplinary focus groups on cascade testing and genetic counseling for fragile X-associated disorders J Genet Couns 2007 16 593 606 10.1007/s10897-007-9099-y 17497108

[B17] Reyniers E Vits L De Boulle K Van Roy B Van Velzen D de Graaff E Verkerk AJ Jorens HZ Darby JK Oostra B The full mutation in the FMR-1 gene of male fragile X patients is absent in their sperm Nat Genet 1993 4 143 146 10.1038/ng0693-143 8348152

[B18] Cronister A Schreiner R Wittenberger M Amiri K Harris K Hagerman RJ Heterozygous fragile X female: historical, physical, cognitive, and cytogenetic features Am J Med Genet 1991 38 269 274 10.1002/ajmg.1320380221 2018071

[B19] Hagerman RJ Leehey M Heinrichs W Tassone F Wilson R Hills J Grigsby J Gage B Hagerman PJ Intention tremor, parkinsonism, and generalized brain atrophy in male carriers of fragile X Neurology 2001 57 127 130 11445641 10.1212/wnl.57.1.127

[B20] Jacquemont S Hagerman RJ Leehey M Grigsby J Zhang L Brunberg JA Greco C Des Portes V Jardini T Levine R Berry-Kravis E Brown WT Schaeffer S Kissel J Tassone F Hagerman PJ Fragile X premutation tremor/ataxia syndrome: molecular, clinical, and neuroimaging correlates Am J Hum Genet 2003 72 869 878 10.1086/374321 12638084 PMC1180350

[B21] Aziz M Stathopulu E Callias M Taylor C Turk J Oostra B Willemsen R Patton M Clinical features of boys with fragile X premutations and intermediate alleles Am J Med Genet B Neuropsychiatr Genet 2003 121 119 127 10.1002/ajmg.b.20030 12898586

[B22] Bourgeois JA Coffey SM Rivera SM Hessl D Gane LW Tassone F Greco C Finucane B Nelson L Berry-Kravis E Grigsby J Hagerman PJ Hagerman RJ A review of fragile X premutation disorders: expanding the psychiatric perspective J Clin Psychiatry 2009 70 852 862 10.4088/JCP.08r04476 19422761 PMC2705685

[B23] Chonchaiya W Schneider A Hagerman RJ Fragile X: a family of disorders Adv Pediatr 2009 56 165 186 10.1016/j.yapd.2009.08.008 19968948 PMC2921504

[B24] Coffey SM Cook K Tartaglia N Tassone F Nguyen DV Pan R Bronsky HE Yuhas J Borodyanskaya M Grigsby J Doerflinger M Hagerman PJ Hagerman RJ Expanded clinical phenotype of women with the FMR1 premutation Am J Med Genet A 2008 146A 1009 1016 10.1002/ajmg.a.32060 18348275 PMC2888464

[B25] Farzin F Perry H Hessl D Loesch D Cohen J Bacalman S Gane L Tassone F Hagerman P Hagerman R Autism spectrum disorders and attention-deficit/hyperactivity disorder in boys with the fragile X premutation J Dev Behav Pediatr 2006 27 S137 S144 10.1097/00004703-200604002-00012 16685180

[B26] Goodlin-Jones B Tassone F Gane LW Hagerman RJ Autistic spectrum disorder and the fragile X premutation J Dev Behav Pediatr 2004 25 392 398 10.1097/00004703-200412000-00002 15613987

[B27] Tassone F Hagerman RJ Taylor AK Gane LW Godfrey TE Hagerman PJ Elevated levels of FMR1 mRNA in carrier males: a new mechanism of involvement in the fragile-X syndrome Am J Hum Genet 2000 66 6 15 10.1086/302720 10631132 PMC1288349

[B28] Garcia-Nonell C Ratera ER Harris S Hessl D Ono MY Tartaglia N Marvin E Tassone F Hagerman RJ Secondary medical diagnosis in fragile X syndrome with and without autism spectrum disorder Am J Med Genet A 2008 146A 1911 1916 10.1002/ajmg.a.32290 18627038 PMC4097171

[B29] Stevens L Tartaglia N Hagerman R Riley K Clinical report: a male with Down syndrome, fragile X syndrome, and autism J Dev Behav Pediatr 31 333 337 10.1097/DBP.0b013e3181d5aa56 20453578 PMC3740577

[B30] Berry-Kravis E Raspa M Loggin-Hester L Bishop E Holiday D D B Epilepsy in fragile X syndrome: characteristics and co-morbid diagnoses Am J Intellect Dev Disabil in press 10.1352/1944-7558-115.6.461 20945999

[B31] Leigh MJ Tassone F Mendoza-Morales G Nguyen D Boyd A Brodovsky J Ruiz C Hessl D Hagerman RJ Evaluation of autism spectrum disorders in females with Fragile X syndrome [abstract] International Meeting for Autism Research; 2010, May 20-22; Philadelphia, PA 2010 375 376 Abstract # 118.169

[B32] Bailey DB Jr Hatton DD Mesibov GB Ament N Skinner M Early development, temperament and functional impairment in autism and fragile X syndrome J Autism Dev Disord 2000 30 49 59 10.1023/A:1005412111706 10819120

[B33] Bailey DB Jr Hatton DD Skinner M Mesibov GB Autistic behavior, FMR1 protein, and developmental trajectories in young males with fragile X syndrome J Autism Dev Disord 2001 31 165 174 10.1023/A:1010747131386 11450815

[B34] Loesch DZ Bui QM Dissanayake C Clifford S Gould E Bulhak-Paterson D Tassone F Taylor AK Hessl D Hagerman R Huggins RM Molecular and cognitive predictors of the continuum of autistic behaviours in fragile X Neurosci Biobehav Rev 2007 31 315 326 10.1016/j.neubiorev.2006.09.007 17097142 PMC2145511

[B35] Lewis P Abbeduto L Murphy M Richmond E Giles N Bruno L Schroeder S Anderson J Orsmond G Psychological well-being of mothers of youth with fragile X syndrome: syndrome specificity and within-syndrome variability J Intellect Disabil Res 2006 50 894 904 10.1111/j.1365-2788.2006.00907.x 17100950

[B36] Hagerman RJ Hagerman RJ, Hagerman PJ Physical and behavioral phenotype Fragile X syndrome: Diagnosis, treatment and research 2002 3 Baltimore: The Johns Hopkins University Press 3 109

[B37] Hessl D Tassone F Cordeiro L Koldewyn K McCormick C Green C Wegelin J Yuhas J Hagerman RJ Brief report: aggression and stereotypic behavior in males with fragile X syndrome--moderating secondary genes in a "single gene" disorder J Autism Dev Disord 2008 38 184 189 10.1007/s10803-007-0365-5 17340199

[B38] Nowicki ST Tassone F Ono MY Ferranti J Croquette MF Goodlin-Jones B Hagerman RJ The Prader-Willi phenotype of fragile X syndrome J Dev Behav Pediatr 2007 28 133 138 10.1097/01.DBP.0000267563.18952.c9 17435464

[B39] Kenet T Froemke RC Schreiner CE Pessah IN Merzenich MM Perinatal exposure to a noncoplanar polychlorinated biphenyl alters tonotopy, receptive fields, and plasticity in rat primary auditory cortex Proc Natl Acad Sci USA 2007 104 7646 7651 10.1073/pnas.0701944104 17460041 PMC1855918

[B40] Waly M Olteanu H Banerjee R Choi SW Mason JB Parker BS Sukumar S Shim S Sharma A Benzecry JM Power-Charnitsky VA Deth RC Activation of methionine synthase by insulin-like growth factor-1 and dopamine: a target for neurodevelopmental toxins and thimerosal Mol Psychiatry 2004 9 358 370 10.1038/sj.mp.4001476 14745455

[B41] Roberts EM English PB Grether JK Windham GC Somberg L Wolff C Maternal residence near agricultural pesticide applications and autism spectrum disorders among children in the California Central Valley Environ Health Perspect 2007 115 1482 1489 17938740 10.1289/ehp.10168 PMC2022638

[B42] Paul R Pessah IN Gane L Ono M Hagerman PJ Brunberg JA Tassone F Bourgeois JA Adams PE Nguyen DV Hagerman R Early onset of neurological symptoms in fragile X premutation carriers exposed to neurotoxins Neurotoxicology 2010 31 399 402 10.1016/j.neuro.2010.04.002 20466021 PMC3918243

[B43] Chen Y Tassone F Berman RF Hagerman PJ Hagerman RJ Willemsen R Pessah IN Murine hippocampal neurons expressing Fmr1 gene premutations show early developmental deficits and late degeneration Hum Mol Genet 2010 19 196 208 10.1093/hmg/ddp479 19846466 PMC2792156

[B44] Ross-Inta C Omanska-Klusek A Wong S Barrow C Garcia-Arocena D Iwahashi C Berry-Kravis E Hagerman RJ Hagerman PJ Giulivi C Mitochondrial dysfunction in fragile X-associated tremor/ataxia syndrome J Biol Chem 2010 429 545 52 10.1042/BJ20091960 PMC4011071 20513237

[B45] O'Dwyer JP Clabby C Crown J Barton DE Hutchinson M Fragile X-associated tremor/ataxia syndrome presenting in a woman after chemotherapy Neurology 2005 65 331 332 10.1212/01.wnl.0000168865.36352.53 16043816

[B46] Qin M Kang J Burlin TV Jiang C Smith CB Postadolescent changes in regional cerebral protein synthesis: an in vivo study in the FMR1 null mouse J Neurosci 2005 25 5087 5095 10.1523/JNEUROSCI.0093-05.2005 15901791 PMC6724856

[B47] Darnell JC van Dreische S Zhang C Mele A Zang JB Fak JJ S-W C Richter J Darnell RB HITS-CLIP identifies specific neuronal mRNA targets of translational repression by the fragile X mental retardation protein, FMRP [abstract] Keystone Symposia; Snowbird, UT 2010 56 Abstract # 016

[B48] Dahlhaus R El-Husseini A Altered neuroligin expression is involved in social deficits in a mouse model of the fragile X syndrome Behav Brain Res 2010 208 96 105 10.1016/j.bbr.2009.11.019 19932134

[B49] Darnell JC Mostovetsky O Darnell RB FMRP RNA targets: identification and validation Genes Brain Behav 2005 4 341 349 10.1111/j.1601-183X.2005.00144.x 16098133

[B50] Miyashiro KY Beckel-Mitchener A Purk TP Becker KG Barret T Liu L Carbonetto S Weiler IJ Greenough WT Eberwine J RNA cargoes associating with FMRP reveal deficits in cellular functioning in Fmr1 null mice Neuron 2003 37 417 431 10.1016/S0896-6273(03)00034-5 12575950

[B51] Butler MG Dasouki MJ Zhou XP Talebizadeh Z Brown M Takahashi TN Miles JH Wang CH Stratton R Pilarski R Eng C Subset of individuals with autism spectrum disorders and extreme macrocephaly associated with germline PTEN tumour suppressor gene mutations J Med Genet 2005 42 318 321 10.1136/jmg.2004.024646 15805158 PMC1736032

[B52] Chiu S Wegelin JA Blank J Jenkins M Day J Hessl D Tassone F Hagerman R Early acceleration of head circumference in children with fragile x syndrome and autism J Dev Behav Pediatr 2007 28 31 35 10.1097/01.DBP.0000257518.60083.2d 17353729

[B53] Nishimura Y Martin CL Vazquez-Lopez A Spence SJ Alvarez-Retuerto AI Sigman M Steindler C Pellegrini S Schanen NC Warren ST Geschwind DH Genome-wide expression profiling of lymphoblastoid cell lines distinguishes different forms of autism and reveals shared pathways Hum Mol Genet 2007 16 1682 1698 10.1093/hmg/ddm116 17519220

[B54] Schenck A Bardoni B Langmann C Harden N Mandel JL Giangrande A CYFIP/Sra-1 controls neuronal connectivity in Drosophila and links the Rac1 GTPase pathway to the fragile X protein Neuron 2003 38 887 898 10.1016/S0896-6273(03)00354-4 12818175

[B55] Sharma A Hoeffer CA Takayasu Y Miyawaki T McBride SM Klann E Zukin RS Dysregulation of mTOR signaling in fragile X syndrome J Neurosci 2010 30 694 702 10.1523/JNEUROSCI.3696-09.2010 20071534 PMC3665010

[B56] Tassone F mTOR up-regulation in patients with FXS [abstract] FRAXA Investigators meeting May 2nd - 5th; Durham, NH 2010

[B57] de Vries PJ Targeted treatments for cognitive and neurodevelopmental disorders in tuberous sclerosis complex Neurotherapeutics 2010 7 275 282 10.1016/j.nurt.2010.05.001 20643380 PMC5084231

[B58] Hazlett HC Poe MD Lightbody AA Gerig G MacFall JR Ross AK Provenzale J Martin A Reiss AL Piven J Teasing apart the heterogeneity of autism: Same behavior, different brains in toddlers with fragile X syndrome and autism J Neurodevelop Disord 2009 1 81 90 10.1007/s11689-009-9009-8 PMC2917990 20700390

[B59] Hernandez RN Feinberg RL Vaurio R Passanante NM Thompson RE Kaufmann WE Autism spectrum disorder in fragile X syndrome: a longitudinal evaluation Am J Med Genet A 2009 149A 1125 1137 10.1002/ajmg.a.32848 19441123 PMC2734278

[B60] Belmonte MK Bourgeron T Fragile X syndrome and autism at the intersection of genetic and neural networks Nat Neurosci 2006 9 1221 1225 10.1038/nn1765 17001341

[B61] Harlow EG Till SM Russell TA Wijetunge LS Kind P Contractor A Critical period plasticity is disrupted in the barrel cortex of FMR1 knockout mice Neuron 65 385 398 10.1016/j.neuron.2010.01.024 20159451 PMC2825250

[B62] Hoeft F Carter JC Lightbody AA Cody Hazlett H Piven J Reiss AL Region-specific alterations in brain development in one- to three-year-old boys with fragile X syndrome Proc Natl Acad Sci USA 2010 107 9335 9339 10.1073/pnas.1002762107 20439717 PMC2889103

[B63] Hagerman RJ Berry-Kravis E Kaufmann WE Ono MY Tartaglia N Lachiewicz A Kronk R Delahunty C Hessl D Visootsak J Picker J Gane L Tranfaglia M Advances in the treatment of fragile X syndrome Pediatrics 2009 123 378 390 10.1542/peds.2008-0317 19117905 PMC2888470

[B64] Amiri K Hagerman RJ Hagerman PJ Fragile X-associated tremor/ataxia syndrome: an aging face of the fragile X gene Arch Neurol 2008 65 19 25 10.1001/archneurol.2007.30 18195136

[B65] Brouwer JR Willemsen R Oostra BA The FMR1 gene and fragile X-associated tremor/ataxia syndrome Am J Med Genet B Neuropsychiatr Genet 2009 150B 782 798 10.1002/ajmg.b.30910 19105204 PMC4320942

[B66] Dick KA Margolis JM Day JW Ranum LP Dominant non-coding repeat expansions in human disease Genome Dyn 2006 1 67 83 full_text 18724054 10.1159/000092501

[B67] Tan H Li H Jin P RNA-mediated pathogenesis in fragile X-associated disorders Neurosci Lett 2009 466 103 108 10.1016/j.neulet.2009.07.053 19631721 PMC2767401

[B68] Garcia-Arocena D Hagerman PJ Advances in understanding the molecular basis of FXTAS Hum Mol Genet 2010 19 R83 89 10.1093/hmg/ddq166 20430935 PMC2875053

[B69] Hagerman PJ Hagerman RJ Fragile X-associated tremor/ataxia syndrome (FXTAS) Ment Retard Dev Disabil Res Rev 2004 10 25 30 10.1002/mrdd.20005 14994285

[B70] Berman RF Willemsen R Mouse models of fragile X-associated tremor ataxia J Investig Med 2009 57 837 841 19574928 10.231/JIM.0b013e3181af59d6 PMC2787904

[B71] Jin P Duan R Qurashi A Qin Y Tian D Rosser TC Liu H Feng Y Warren ST Pur alpha binds to rCGG repeats and modulates repeat-mediated neurodegeneration in a Drosophila model of fragile X tremor/ataxia syndrome Neuron 2007 55 556 564 10.1016/j.neuron.2007.07.020 17698009 PMC1994817

[B72] Sofola OA Jin P Qin Y Duan R Liu H de Haro M Nelson DL Botas J RNA-binding proteins hnRNP A2/B1 and CUGBP1 suppress fragile X CGG premutation repeat-induced neurodegeneration in a Drosophila model of FXTAS Neuron 2007 55 565 571 10.1016/j.neuron.2007.07.021 17698010 PMC2215388

[B73] Willemsen R Hoogeveen-Westerveld M Reis S Holstege J Severijnen LA Nieuwenhuizen IM Schrier M van Unen L Tassone F Hoogeveen AT Hagerman PJ Mientjes EJ Oostra BA The FMR1 CGG repeat mouse displays ubiquitin-positive intranuclear neuronal inclusions; implications for the cerebellar tremor/ataxia syndrome Hum Mol Genet 2003 12 949 959 10.1093/hmg/ddg114 12700164

[B74] Entezam A Biacsi R Orrison B Saha T Hoffman GE Grabczyk E Nussbaum RL Usdin K Regional FMRP deficits and large repeat expansions into the full mutation range in a new Fragile X premutation mouse model Gene 2007 395 125 134 10.1016/j.gene.2007.02.026 17442505 PMC1950257

[B75] Handa V Saha T Usdin K The fragile X syndrome repeats form RNA hairpins that do not activate the interferon-inducible protein kinase, PKR, but are cut by Dicer Nucleic Acids Res 2003 31 6243 6248 10.1093/nar/gkg818 14576312 PMC275460

[B76] Hashem V Galloway JN Mori M Willemsen R Oostra BA Paylor R Nelson DL Ectopic expression of CGG containing mRNA is neurotoxic in mammals Hum Mol Genet 2009 18 2443 2451 10.1093/hmg/ddp182 19377084 PMC2694692

[B77] Jin P Zarnescu DC Zhang F Pearson CE Lucchesi JC Moses K Warren ST RNA-mediated neurodegeneration caused by the fragile X premutation rCGG repeats in *Drosophila* Neuron 2003 39 739 747 10.1016/S0896-6273(03)00533-6 12948442

[B78] Sofola OA Jin P Botas J Nelson DL Argonaute-2-dependent rescue of a Drosophila model of FXTAS by FRAXE premutation repeat Hum Mol Genet 2007 16 2326 2332 10.1093/hmg/ddm186 17635840

[B79] Van Dam D Errijgers V Kooy RF Willemsen R Mientjes E Oostra BA De Deyn PP Cognitive decline, neuromotor and behavioural disturbances in a mouse model for fragile-X-associated tremor/ataxia syndrome (FXTAS) Behav Brain Res 2005 162 233 239 10.1016/j.bbr.2005.03.007 15876460

[B80] Allen E He W Yadav-Shah M Sherman S A study of the distributional characteristics of *FMR1 *transcript levels in 238 individuals Human Genetics 2004 114 439 447 10.1007/s00439-004-1086-x 14758538

[B81] Kenneson A Zhang F Hagedorn CH Warren ST Reduced FMRP and increased FMR1 transcription is proportionally associated with CGG repeat number in intermediate-length and premutation carriers Hum Mol Genet 2001 10 1449 1454 10.1093/hmg/10.14.1449 11448936

[B82] Hunter JE Rohr JK Sherman SL Co-occurring diagnoses among FMR1 premutation allele carriers Clin Genet 2010 77 374 381 10.1111/j.1399-0004.2009.01317.x 20059484 PMC3696492

[B83] Sullivan AK Marcus M Epstein MP Allen EG Anido AE Paquin JJ Yadav-Shah M Sherman SL Association of FMR1 repeat size with ovarian dysfunction Hum Reprod 2005 20 402 412 10.1093/humrep/deh635 15608041

[B84] Wittenberger MD Hagerman RJ Sherman SL McConkie-Rosell A Welt CK Rebar RW Corrigan EC Simpson JL Nelson LM The FMR1 premutation and reproduction Fertil Steril 2007 87 456 465 10.1016/j.fertnstert.2006.09.004 17074338

[B85] Greco CM Hagerman RJ Tassone F Chudley AE Del Bigio MR Jacquemont S Leehey M Hagerman PJ Neuronal intranuclear inclusions in a new cerebellar tremor/ataxia syndrome among fragile X carriers Brain 2002 125 1760 1771 10.1093/brain/awf184 12135967

[B86] Tassone F Iwahashi C Hagerman PJ FMR1 RNA within the intranuclear inclusions of fragile X-associated tremor/ataxia syndrome (FXTAS) RNA Biol 2004 1 103 105 17179750 10.4161/rna.1.2.1035

[B87] Bailey DB Jr Raspa M Olmsted M Holiday DB Co-occurring conditions associated with FMR1 gene variations: findings from a national parent survey Am J Med Genet A 2008 146A 2060 2069 10.1002/ajmg.a.32439 18570292

[B88] Garcia-Arocena D Yang JE Brouwer JR Tassone F Iwahashi C Berry-Kravis EM Goetz CG Sumis AM Zhou L Nguyen DV Campos L Howell E Ludwig A Greco C Willemsen R Hagerman RJ Hagerman PJ Fibroblast phenotype in male carriers of *FMR1 *premutation alleles Hum Mol Genet 2010 19 299 312 10.1093/hmg/ddp497 19864489 PMC2796892

[B89] Hunsaker MR Wenzel HJ Willemsen R Berman RF Progressive spatial processing deficits in a mouse model of the fragile X premutation Behav Neurosci 2009 123 1315 1324 10.1037/a0017616 20001115 PMC3410547

[B90] Tassone F Adams J Berry-Kravis EM Cohen SS Brusco A Leehey MA Li L Hagerman RJ Hagerman PJ CGG repeat length correlates with age of onset of motor signs of the fragile X-associated tremor/ataxia syndrome (FXTAS) Am J Med Genet B Neuropsychiatr Genet 2007 144 566 569 10.1002/ajmg.b.30482 17427188

[B91] Lee JE Cooper TA Pathogenic mechanisms of myotonic dystrophy Biochem Soc Trans 2009 37 1281 1286 10.1042/BST0371281 19909263 PMC3873089

[B92] Wheeler TM Thornton CA Myotonic dystrophy: RNA-mediated muscle disease Curr Opin Neurol 2007 20 572 576 10.1097/WCO.0b013e3282ef6064 17885447

[B93] Arocena DG Iwahashi CK Won N Beilina A Ludwig AL Tassone F Schwartz PH Hagerman PJ Induction of inclusion formation and disruption of lamin A/C structure by premutation CGG-repeat RNA in human cultured neural cells Hum Mol Genet 2005 14 3661 3671 10.1093/hmg/ddi394 16239243

[B94] Sellier C Rau F Liu Y Tassone F Hukema RK Gattoni R Schneider A Richard S Willemsen R Elliott DJ Hagerman PJ Charlet-Berguerand N Sam68 sequestration and partial loss of function are associated with splicing alterations in FXTAS patients EMBO J 2010 29 1248 1261 10.1038/emboj.2010.21 20186122 PMC2857464

[B95] Sellier C Hagerman P Willemsen R Charlet-Berguerand N DROSHA/DGCR8 sequestration by expanded CGG repeats leads to global micro-RNA processing alteration in FXTAS patients [abstract] 12th International Fragile X Conference; July 21-25; Detroit, MI 2010

[B96] Mah LJ El-Osta A Karagiannis TC gammaH2AX: a sensitive molecular marker of DNA damage and repair Leukemia 2010 24 679 686 10.1038/leu.2010.6 20130602

[B97] Iwahashi CK Yasui DH An HJ Greco CM Tassone F Nannen K Babineau B Lebrilla CB Hagerman RJ Hagerman PJ Protein composition of the intranuclear inclusions of FXTAS Brain 2006 129 256 271 10.1093/brain/awh650 16246864

[B98] Oliveira G Diogo L Grazina M Garcia P Ataide A Marques C Miguel T Borges L Vicente AM Oliveira CR Mitochondrial dysfunction in autism spectrum disorders: a population-based study Dev Med Child Neurol 2005 47 185 189 10.1017/S0012162205000332 15739723

[B99] Weissman JR Kelley RI Bauman ML Cohen BH Murray KF Mitchell RL Kern RL Natowicz MR Mitochondrial disease in autism spectrum disorder patients: a cohort analysis PLoS One 2008 3 e3815 10.1371/journal.pone.0003815 19043581 PMC2584230

[B100] Graf WD Marin-Garcia J Gao HG Pizzo S Naviaux RK Markusic D Barshop BA Courchesne E Haas RH Autism associated with the mitochondrial DNA G8363A transfer RNA(Lys) mutation J Child Neurol 2000 15 357 361 10.1177/088307380001500601 10868777

[B101] Pons R Andreu AL Checcarelli N Vila MR Engelstad K Sue CM Shungu D Haggerty R de Vivo DC DiMauro S Mitochondrial DNA abnormalities and autistic spectrum disorders J Pediatr 2004 144 81 85 10.1016/j.jpeds.2003.10.023 14722523

[B102] Filipek PA Juranek J Smith M Mays LZ Ramos ER Bocian M Masser-Frye D Laulhere TM Modahl C Spence MA Gargus JJ Mitochondrial dysfunction in autistic patients with 15q inverted duplication Ann Neurol 2003 53 801 804 10.1002/ana.10596 12783428

[B103] Hagerman RJ Staley LW O'Connor R Lugenbeel K Nelson D McLean SD Taylor A Learning-disabled males with a fragile X CGG expansion in the upper premutation size range Pediatrics 1996 97 122 126 8545206

[B104] Loesch DZ Hay DA Mulley J Transmitting males and carrier females in fragile X--revisited Am J Med Genet 1994 51 392 399 10.1002/ajmg.1320510418 7943005

[B105] Tassone F Hagerman RJ Taylor AK Mills JB Harris SW Gane LW Hagerman PJ Clinical involvement and protein expression in individuals with the FMR1 premutation Am J Med Genet 2000 91 144 152 10.1002/(SICI)1096-8628(20000313)91:2<144::AID-AJMG14>3.0.CO;2-V 10748416

[B106] Aziz M Stathopulu E Callias M Taylor C Turk J Oostra B Willemsen R Patton M Clinical features of boys with fragile X premutations and intermediate alleles Am J Med Genet 2003 121B 119 127 10.1002/ajmg.b.20030 12898586

[B107] Clifford S Dissanayake C Bui QM Huggins R Taylor AK Loesch DZ Autism spectrum phenotype in males and females with fragile X full mutation and premutation J Autism Dev Disord 2007 37 738 747 10.1007/s10803-006-0205-z 17031449

[B108] Farzin F Perry H Hessl D Loesch D Cohen J Bacalman S Gane L Tassone F Hagerman P Hagerman R Autism spectrum disorders and attention-deficit/hyperactivity disorder in boys with the fragile X premutation J Dev Behav Pediatr 2006 27 S137 144 10.1097/00004703-200604002-00012 16685180

[B109] Loesch DZ Cook M Litewka L Gould E Churchyard A Tassone F Slater HR Storey E A low symptomatic form of neurodegeneration in younger carriers of the FMR1 premutation, manifesting typical radiological changes J Med Genet 2008 45 179 181 10.1136/jmg.2007.054171 18057083

[B110] Hashimoto RI Backer KC Tassone F Hagerman RJ Rivera SM An fMRI study of the prefrontal activity during the performance of a working memory task in premutation carriers of the fragile X mental retardation 1 gene with and without fragile X-associated tremor/ataxia syndrome (FXTAS) J Psychiatr Res 2010 20537351 10.1016/j.jpsychires.2010.04.030 PMC2978252

[B111] Hashimoto R Srivastava S Tassone F Hagerman RJ Rivera SM Diffusion tensor imaging in male premutation carriers of the fragile X mental retardation gene Movement Disord in press 10.1002/mds.23646 PMC3119762 21484870

[B112] Loesch DZ Bui QM Grigsby J Butler E Epstein J Huggins RM Taylor AK Hagerman RJ Effect of the fragile X status categories and the fragile X mental retardation protein levels on executive functioning in males and females with fragile X Neuropsychology 2003 17 646 657 10.1037/0894-4105.17.4.646 14599277

[B113] Grigsby J Brega AG Engle K Leehey MA Hagerman RJ Tassone F Hessl D Hagerman PJ Cogswell JB Bennett RE Cook K Hall DA Bounds LS Paulich MJ Reynolds A Cognitive profile of fragile X premutation carriers with and without fragile X-associated tremor/ataxia syndrome Neuropsychology 2008 22 48 60 10.1037/0894-4105.22.1.48 18211155

[B114] Moore CJ Daly EM Schmitz N Tassone F Tysoe C Hagerman RJ Hagerman PJ Morris RG Murphy KC Murphy DG A neuropsychological investigation of male premutation carriers of fragile X syndrome Neuropsychologia 2004 42 1934 1947 10.1016/j.neuropsychologia.2004.05.002 15381024

[B115] Cornish KM Li L Kogan CS Jacquemont S Turk J Dalton A Hagerman RJ Hagerman PJ Age-dependent cognitive changes in carriers of the fragile X syndrome Cortex 2008 44 628 636 10.1016/j.cortex.2006.11.002 18472033 PMC11060834

[B116] Cornish KM Kogan CS Li L Turk J Jacquemont S Hagerman RJ Lifespan changes in working memory in fragile X premutation males Brain Cogn 2009 69 551 558 10.1016/j.bandc.2008.11.006 19114290 PMC4158922

[B117] Kogan CS Cornish KM Mapping self-reports of working memory deficits to executive dysfunction in fragile X mental retardation 1 (FMR1) gene premutation carriers asymptomatic for FXTAS Brain Cogn 2010 73 236 243 10.1016/j.bandc.2010.05.008 20573435

[B118] Hunter JE Allen EG Abramowitz A Rusin M Leslie M Novak G Hamilton D Shubeck L Charen K Sherman SL No evidence for a difference in neuropsychological profile among carriers and noncarriers of the FMR1 premutation in adults under the age of 50 Am J Hum Genet 2008 83 692 702 10.1016/j.ajhg.2008.10.021 19026394 PMC2668066

[B119] Grigsby J Hills J Wilson R Leehey M Hagerman RJ Tassone F Hagerman PJ Dysexecutive syndrome in older men with action tremor and the fragile X premutation J Int Neuropsychol Soc 2002 8 282

[B120] Roberts J Mazzocco MM Murphy MM Hoehn-Saric R Arousal modulation in females with fragile X or Turner syndrome J Autism Dev Disord 2008 38 20 27 10.1007/s10803-007-0356-6 17340202 PMC2730938

[B121] Rodriguez-Revenga L Madrigal I Alegret M Santos M Mila M Evidence of depressive symptoms in fragile-X syndrome premutated females Psychiatr Genet 2008 18 153 155 10.1097/YPG.0b013e3282f97e0b 18628675

[B122] Franke P Leboyer M Gansicke M Weiffenbach O Biancalana V Cornillet-Lefebre P Croquette MF Froster U Schwab SG Poustka F Hautzinger M Maier W Genotype-phenotype relationship in female carriers of the premutation and full mutation of FMR-1 Psychiatry Res 1998 80 113 127 10.1016/S0165-1781(98)00055-9 9754690

[B123] Hessl D Tassone F Loesch DZ Berry-Kravis E Leehey MA Gane LW Barbato I Rice C Gould E Hall DA Grigsby J Wegelin JA Harris S Lewin F Weinberg D Hagerman PJ Hagerman RJ Abnormal elevation of FMR1 mRNA is associated with psychological symptoms in individuals with the fragile X premutation Am J Med Genet B Neuropsychiatr Genet 2005 139B 115 121 10.1002/ajmg.b.30241 16184602

[B124] Bourgeois J Seritan A Casillas E Hessl D Schneider A Yang Y Kaur I Cogswell J Nguyen D Hagerman R Lifetime prevalence of mood and anxiety disorders in fragile X premutation carriers J Clin Psychiatry 2010 10.4088/JCP.4009m05407blu 20816038 10.4088/JCP.09m05407blu PMC4038118

[B125] Rodriguez-Revenga L Madrigal I Pagonabarraga J Xuncla M Badenas C Kulisevsky J Gomez B Mila M Penetrance of FMR1 premutation associated pathologies in fragile X syndrome families Eur J Hum Genet 2009 17 1359 1362 10.1038/ejhg.2009.51 19367323 PMC2986640

[B126] Zhang L Coffey S Lua LL Greco CM Schafer JA Brunberg J Borodyanskaya M Agius MA Apperson M Leehey M Tartaglia N Tassone F Hagerman PJ Hagerman RJ FMR1 premutation in females diagnosed with multiple sclerosis J Neurol Neurosurg Psychiatry 2009 80 812 814 10.1136/jnnp.2008.160960 19531693

[B127] Greco CM Tassone F Garcia-Arocena D Tartaglia N Coffey SM Vartanian TK Brunberg JA Hagerman PJ Hagerman RJ Clinical and neuropathologic findings in a woman with the FMR1 premutation and multiple sclerosis Arch Neurol 2008 65 1114 1116 10.1001/archneur.65.8.1114 18695063 PMC3081275

[B128] Chonchaiya W Tassone F Ashwood P Hessl D Schneider A Campos L Nguyen D Au J Hagerman R Autoimmune disease in mothers with the FMR1 premutation is associated with seizures in their children with fragile X syndrome J Hum Genet in press 10.1007/s00439-010-0882-8 PMC2955238 20809278

[B129] Zalfa F Eleuteri B Dickson KS Mercaldo V De Rubeis S di Penta A Tabolacci E Chiurazzi P Neri G Grant SG Bagni C A new function for the fragile X mental retardation protein in regulation of PSD-95 mRNA stability Nat Neurosci 2007 10 578 587 10.1038/nn1893 17417632 PMC2804293

[B130] Penagarikano O Mulle JG Warren ST The pathophysiology of fragile X syndrome Annu Rev Genomics Hum Genet 2007 8 109 129 10.1146/annurev.genom.8.080706.092249 17477822

[B131] Bassell GJ Warren ST Fragile X syndrome: loss of local mRNA regulation alters synaptic development and function Neuron 2008 60 201 214 10.1016/j.neuron.2008.10.004 18957214 PMC3691995

[B132] Luo Y Shan G Guo W Smrt RD Johnson EB Li X Pfeiffer RL Szulwach KE Duan R Barkho BZ Li W Liu C Jin P Zhao X Fragile x mental retardation protein regulates proliferation and differentiation of adult neural stem/progenitor cells PLoS Genet 2010 6 e1000898 10.1371/journal.pgen.1000898 20386739 PMC2851565

[B133] Utari A Adams E Berry-Kravis E Chavez A Scaggs F Ngotran L Boyd A Hessl D Gane LW Tassone F Tartaglia N Leehey MA Hagerman RJ Aging in fragile X syndrome J Neurodev Disord 2010 2 70 76 10.1007/s11689-010-9047-2 20585378 PMC2882562

[B134] Wegiel J Kuchna I Nowicki K Imaki H Wegiel J Marchi E Ma SY Chauhan A Chauhan V Bobrowicz TW de Leon M Louis LA Cohen IL London E Brown WT Wisniewski T The neuropathology of autism: defects of neurogenesis and neuronal migration, and dysplastic changes Acta Neuropathol 2010 119 755 770 10.1007/s00401-010-0655-4 20198484 PMC2869041

[B135] Bear MF Huber KM Warren ST The mGluR theory of fragile X mental retardation Trends Neurosci 2004 27 370 377 10.1016/j.tins.2004.04.009 15219735

[B136] Dolen G Bear MF Role for metabotropic glutamate receptor 5 (mGluR5) in the pathogenesis of fragile X syndrome J Physiol 2008 586 1503 1508 10.1113/jphysiol.2008.150722 18202092 PMC2375688

[B137] Comery TA Harris JB Willems PJ Oostra BA Irwin SA Weiler IJ Greenough WT Abnormal dendritic spines in fragile X knockout mice: maturation and pruning deficits Proc Natl Acad Sci USA 1997 94 5401 5404 10.1073/pnas.94.10.5401 9144249 PMC24690

[B138] Nimchinsky EA Oberlander AM Svoboda K Abnormal development of dendritic spines in FMR1 knock-out mice J Neurosci 2001 21 5139 5146 11438589 10.1523/JNEUROSCI.21-14-05139.2001 PMC6762842

[B139] Irwin SA Patel B Idupulapati M Harris JB Crisostomo RA Larsen BP Kooy F Willems PJ Cras P Kozlowski PB Swain RA Weiler IJ Greenough WT Abnormal dendritic spine characteristics in the temporal and visual cortices of patients with fragile-X syndrome: a quantitative examination Am J Med Genet 2001 98 161 167 10.1002/1096-8628(20010115)98:2<161::AID-AJMG1025>3.0.CO;2-B 11223852

[B140] Bilousova TV Dansie L Ngo M Aye J Charles JR Ethell DW Ethell IM Minocycline promotes dendritic spine maturation and improves behavioural performance in the fragile X mouse model J Med Genet 2009 46 94 102 10.1136/jmg.2008.061796 18835858

[B141] Hinton VJ Brown WT Wisniewski K Rudelli RD Analysis of neocortex in three males with the fragile X syndrome Am J Med Genet 1991 41 289 294 10.1002/ajmg.1320410306 1724112

[B142] Irwin SA Galvez R Greenough WT Dendritic spine structural anomalies in fragile-X mental retardation syndrome Cerebral Cortex 2000 10 1038 1044 10.1093/cercor/10.10.1038 11007554

[B143] Chuang SC Zhao W Bauchwitz R Yan Q Bianchi R Wong RK Prolonged epileptiform discharges induced by altered group I metabotropic glutamate receptor-mediated synaptic responses in hippocampal slices of a fragile X mouse model J Neurosci 2005 25 8048 8055 10.1523/JNEUROSCI.1777-05.2005 16135762 PMC6725444

[B144] Berry-Kravis E Epilepsy in fragile X syndrome Developmental Medicine and Child Neurology 2002 44 724 728 10.1111/j.1469-8749.2002.tb00277.x 12418611

[B145] Dolen G Osterweil E Rao BS Smith GB Auerbach BD Chattarji S Bear MF Correction of fragile X syndrome in mice Neuron 2007 56 955 962 10.1016/j.neuron.2007.12.001 18093519 PMC2199268

[B146] Bear MF Therapeutic implications of the mGluR theory of fragile X mental retardation Genes Brain Behav 2005 4 393 398 10.1111/j.1601-183X.2005.00135.x 16098137

[B147] Gasparini F Lingenhohl K Stoehr N Flor PJ Heinrich M Vranesic I Biollaz M Allgeier H Heckendorn R Urwyler S Varney MA Johnson EC Hess SD Rao SP Sacaan AI Santori EM Veliçelebi G Kuhn R 2-Methyl-6-(phenylethynyl)-pyridine (MPEP), a potent, selective and systemically active mGlu5 receptor antagonist Neuropharmacology 1999 38 1493 1503 10.1016/S0028-3908(99)00082-9 10530811

[B148] Yan QJ Rammal M Tranfaglia M Bauchwitz RP Suppression of two major fragile X syndrome mouse model phenotypes by the mGluR5 antagonist MPEP Neuropharmacology 2005 49 1053 1066 10.1016/j.neuropharm.2005.06.004 16054174

[B149] de Vrij FM Levenga J van der Linde HC Koekkoek SK De Zeeuw CI Nelson DL Oostra BA Willemsen R Rescue of behavioral phenotype and neuronal protrusion morphology in Fmr1 KO mice Neurobiol Dis 2008 31 127 132 10.1016/j.nbd.2008.04.002 18571098 PMC2481236

[B150] Bauchwitz RP Yan Q Rammal M Modulation of mGluR5 activity *in vivo *can ameliorate phenotypic markers of fragile X syndrome in mice Society for Neuroscience; October 23-27; San Diego, CA 2004 Society for Neuroscience 583.520

[B151] McBride SM Choi CH Wang Y Liebelt D Braunstein E Ferreiro D Sehgal A Siwicki KK Dockendorff TC Nguyen HT McDonald TV Jongens TA Pharmacological rescue of synaptic plasticity, courtship behavior, and mushroom body defects in a Drosophila model of fragile X syndrome Neuron 2005 45 753 764 10.1016/j.neuron.2005.01.038 15748850

[B152] Berry-Kravis E Hessl D Coffey S Hervey C Schneider A Yuhas J Hutchison J Snape M Tranfaglia M Nguyen DV Hagerman R A pilot open label, single dose trial of fenobam in adults with fragile X syndrome J Med Genet 2009 46 266 271 10.1136/jmg.2008.063701 19126569 PMC2658751

[B153] Wang LW Berry-Kravis E Hagerman RJ Fragile X: leading the way for targeted treatments in autism Neurotherapeutics 2010 7 264 274 10.1016/j.nurt.2010.05.005 20643379 PMC4084556

[B154] Isaacson JS Hille B GABA(B)-mediated presynaptic inhibition of excitatory transmission and synaptic vesicle dynamics in cultured hippocampal neurons Neuron 1997 18 143 152 10.1016/S0896-6273(01)80053-2 9010212

[B155] Scanziani M Capogna M Gahwiler BH Thompson SM Presynaptic inhibition of miniature excitatory synaptic currents by baclofen and adenosine in the hippocampus Neuron 1992 9 919 927 10.1016/0896-6273(92)90244-8 1358131

[B156] Sohn JW Lee D Cho H Lim W Shin HS Lee SH Ho WK Receptor-specific inhibition of GABAB-activated K+ currents by muscarinic and metabotropic glutamate receptors in immature rat hippocampus J Physiol 2007 580 411 422 10.1113/jphysiol.2006.125914 17255165 PMC2075565

[B157] Zupan B Toth M Inactivation of the maternal fragile X gene results in sensitization of GABAB receptor function in the offspring J Pharmacol Exp Ther 2008 jpet.108.143990 18812493 10.1124/jpet.108.143990 PMC2666962

[B158] Pacey LK Heximer SP Hampson DR Increased GABA(B) receptor-mediated signaling reduces the susceptibility of fragile X knockout mice to audiogenic seizures Mol Pharmacol 2009 76 18 24 10.1124/mol.109.056127 19351745

[B159] Berry-Kravis E Cherubini M Zarevics P Rathmell B Wang PP Carpenter R Bear M Hagerman R Arbaclofen for the treatment of children and adults with fragile X syndrome: results of a phase 2, randomized, double-blind, placebo-controlled, crossover study [Abstract] International Meeting for Autism Research; Philadelphia, PA 2010 741 Abstract # 140.004

[B160] Mihalek RM Banerjee PK Korpi ER Quinlan JJ Firestone LL Mi ZP Lagenaur C Tretter V Sieghart W Anagnostaras SG Sage JR Fanselow MS Guidotti A Spigelman I Li Z DeLorey TM Olsen RW Homanics GE Attenuated sensitivity to neuroactive steroids in gamma-aminobutyrate type A receptor delta subunit knockout mice Proc Natl Acad Sci USA 1999 96 12905 12910 10.1073/pnas.96.22.12905 10536021 PMC23157

[B161] Carter RB Wood PL Wieland S Hawkinson JE Belelli D Lambert JJ White HS Wolf HH Mirsadeghi S Tahir SH Bolger MB Lan NC Gee KW Characterization of the anticonvulsant properties of ganaxolone (CCD 1042; 3alpha-hydroxy-3beta-methyl-5alpha-pregnan-20-one), a selective, high-affinity, steroid modulator of the gamma-aminobutyric acid(A) receptor J Pharmacol Exp Ther 1997 280 1284 1295 9067315

[B162] Bormann J The 'ABC' of GABA receptors Trends Pharmacol Sci 2000 21 16 19 10.1016/S0165-6147(99)01413-3 10637650

[B163] Chebib M Johnston GA The 'ABC' of GABA receptors: a brief review Clin Exp Pharmacol Physiol 1999 26 937 940 10.1046/j.1440-1681.1999.03151.x 10561820

[B164] D'Hulst C Kooy RF The GABAA receptor: a novel target for treatment of fragile X? Trends Neurosci 2007 30 425 431 10.1016/j.tins.2007.06.003 17590448

[B165] Miyashiro KY Beckel-Mitchener A Purk TP Becker KG Barret T Liu L Carbonetto S Weiler IJ Greenough WT Eberwine J RNA cargoes associating with FMRP reveal deficits in cellular functioning in Fmr1 null mice Neuron 2003 37 417 431 10.1016/S0896-6273(03)00034-5 12575950

[B166] Chang S Bray SM Li Z Zarnescu DC He C Jin P Warren ST Identification of small molecules rescuing fragile X syndrome phenotypes in Drosophila Nat Chem Biol 2008 4 256 263 10.1038/nchembio.78 18327252

[B167] D'Hulst C De Geest N Reeve SP Van Dam D De Deyn PP Hassan BA Kooy RF Decreased expression of the GABAA receptor in fragile X syndrome Brain Res 2006 1121 238 245 10.1016/j.brainres.2006.08.115 17046729

[B168] Gantois I Vandesompele J Speleman F Reyniers E D'Hooge R Severijnen LA Willemsen R Tassone F Kooy RF Expression profiling suggests underexpression of the GABA(A) receptor subunit delta in the fragile X knockout mouse model Neurobiol Dis 2006 21 346 357 10.1016/j.nbd.2005.07.017 16199166

[B169] Selby L Zhang C Sun QQ Major defects in neocortical GABAergic inhibitory circuits in mice lacking the fragile X mental retardation protein Neurosci Lett 2007 412 227 232 10.1016/j.neulet.2006.11.062 17197085 PMC1839948

[B170] D'Hulst C Heulens I Brouwer JR Willemsen R De Geest N Reeve SP De Deyn PP Hassan BA Kooy RF Expression of the GABAergic system in animal models for fragile X syndrome and fragile X associated tremor/ataxia syndrome (FXTAS) Brain Research 2009 1253 176 183 10.1016/j.brainres.2008.11.075 19070606

[B171] Curia G Papouin T Seguela P Avoli M Downregulation of tonic GABAergic inhibition in a mouse model of fragile X syndrome Cereb Cortex 2009 19 1515 1520 10.1093/cercor/bhn159 18787232 PMC4873279

[B172] El Idrissi A Ding XH Scalia J Trenkner E Brown WT Dobkin C Decreased GABA(A) receptor expression in the seizure-prone fragile X mouse Neurosci Lett 2005 377 141 146 10.1016/j.neulet.2004.11.087 15755515

[B173] Kooy RF Of mice and the fragile X syndrome Trends Genet 2003 19 148 154 10.1016/S0168-9525(03)00017-9 12615009

[B174] D'Antuono M Merlo D Avoli M Involvement of cholinergic and gabaergic systems in the fragile X knockout mice Neuroscience 2003 119 9 13 10.1016/S0306-4522(03)00103-9 12763063

[B175] Kooy F Heulens I D'Hulst C Van der Aa N Bagni C Hassan B De Deyn P The GABAA receptor as a potential target for therapy of the fragile X syndrome [abstract] NFXF 12th International FX Conference; July 21 - 25; Detroit, MI 2010

[B176] Reddy DS Pharmacology of endogenous neuroactive steroids Crit Rev Neurobiol 2003 15 197 234 10.1615/CritRevNeurobiol.v15.i34.20 15248811

[B177] Bialer M Johannessen SI Kupferberg HJ Levy RH Perucca E Tomson T Progress report on new antiepileptic drugs: a summary of the Eighth Eilat Conference (EILAT VIII) Epilepsy Res 2007 73 1 52 10.1016/j.eplepsyres.2006.10.008 17158031

[B178] Rogawski MA Reddy DS Rho JM, Sankar R, Cavazos J Neurosteroids: endogenous modulators of seizure susceptibility Epilepsy: Scientific Foundations of Clinical Practice 2004 New York: Marcel Dekker 319 355

[B179] Laxer K Blum D Abou-Khalil BW Morrell MJ Lee DA Data JL Monaghan EP Assessment of ganaxolone's anticonvulsant activity using a randomized, double-blind, presurgical trial design. Ganaxolone Presurgical Study Group Epilepsia 2000 41 1187 1194 10.1111/j.1528-1157.2000.tb00324.x 10999558

[B180] Kerrigan JF Shields WD Nelson TY Bluestone DL Dodson WE Bourgeois BF Pellock JM Morton LD Monaghan EP Ganaxolone for treating intractable infantile spasms: a multicenter, open-label, add-on trial Epilepsy Res 2000 42 133 139 10.1016/S0920-1211(00)00170-4 11074186

[B181] Bilousova TV Rusakov DA Ethell DW Ethell IM Matrix metalloproteinase-7 disrupts dendritic spines in hippocampal neurons through NMDA receptor activation J Neurochem 2006 97 44 56 10.1111/j.1471-4159.2006.03701.x 16515559 PMC3369267

[B182] Utari A Chonchaiya W Rivera SM Schneider A Hagerman RJ Faradz SM Ethell IM Nguyen DV Side effects of minocycline treatment in patients with fragile X syndrome and exploration of outcome measures Am J Intellect Dev Disabil 2010 115 433 443 20687826 10.1352/1944-7558-115.5.433 PMC4031088

[B183] Paribello Open label add on treatment trial of minocycline in patients with fragile X syndrome [abstract] FRAXA Investigators Meeting May 2nd - 5th; Durham, NH 2010 10.1186/1471-2377-10-91 PMC2958860 20937127

